# Unveiling the role of the venous system in arterial hypertension: A computational study

**DOI:** 10.1113/JP289895

**Published:** 2026-07-31

**Authors:** Eleuterio F. Toro, Lucas O. Müller, Tonja W. Emans, Fiona D. McBryde, Julian F. R. Paton

**Affiliations:** ^1^ Laboratory of Applied Mathematics, Department of Civil, Environmental and Mechanical Engineering University of Trento Trento Italy; ^2^ Laboratory of Mathematics for Biology and Medicine, Department of Mathematics University of Trento Trento Italy; ^3^ The Centre for Heart Research, Department of Physiology, Faculty of Medical and Health Sciences University of Auckland Auckland New Zealand

**Keywords:** arterial haemodynamics, hypertension, mathematical model, venous haemodynamics

## Abstract

**Abstract:**

Arterial hypertension is a prevalent condition and a major risk factor for cardiovascular and neurological diseases. Notably, medical treatment fails to control blood pressure in a large proportion of diagnosed subjects, highlighting gaps in our understanding of hypertension's pathophysiology. Using a mathematical model of the human cardiovascular system, we quantify the contributions of various cardiovascular compartments to the hypertensive state. The impact of uncertainty in modelling assumptions is assessed through stochastic modelling. Our results show that reduced venous vascular compliance significantly increases arterial pressure, making it the second most influential factor in determining hypertension, after increased peripheral resistance.

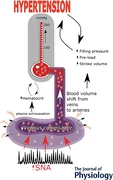

**Key points:**

- We used a closed‐loop mathematical model of the full human circulation to simulate adaptation to hypertension, including stochastic variation in parameters.
- The model quantifies the relative importance of different cardiovascular parameters in determining arterial pressure.
- Venous compliance emerged as the second most influential determinant of arterial pressure, after systemic resistance.
- Reduced venous compliance alone was sufficient to cause a hypertensive state in the model.
- These findings highlight the potential of the venous system as a novel therapeutic target for blood pressure regulation.

## Graphical abstract

**Abstract figure legend** This abstract figure summarizes the principal findings of our computational analysis of hypertension. Model‐based results identify reduced venous compliance as a major determinant of elevated arterial pressure, second only to increased peripheral resistance. Decreased venous capacitance promotes a redistribution of blood volume from the venous reservoir to the arterial circulation, increasing cardiac filling pressure, preload and stroke volume. Building on these findings the schematic highlights physiological mechanisms discussed in the manuscript that may contribute to this behaviour, including enhanced sympathetic nerve activity, plasma extravasation and changes in haematocrit. Together these results underscore the central role of the venous system in the development of hypertension and emphasize its importance alongside arterial factors in determining systemic haemodynamics.

## Introduction

Hypertension‐related complications account for over 10 million deaths per year worldwide. As shown in the recent study by Zhou et al. (2021), in 2019, more than 1.28 billion adults aged 30–79 were affected by hypertension, with a global age‐standardized prevalence of 34% and 32% for men and women, respectively. These numbers clearly show the relevance of hypertension as a global medical condition. Furthermore, the alluded study also shows that just over one half of hypertensives were diagnosed. Even more revealing, of those diagnosed and treated, less than one half responded positively to therapy. This indicates that the basic mechanisms of essential hypertension onset and development are not fully understood.

Although the aetiology of secondary hypertension types, such as those related to renal pathologies or obstructive sleep apnoea, is well known and documented (Rimoldi et al., 2014; Rossi et al., 2020), there is currently a debate regarding the triggering mechanisms in the onset and development of hypertension. Because essential hypertension manifests in terms of alterations/adaptations of the cardiovascular system, treatment has focused on some of these manifestations. For example, beta‐blockers lower blood pressure by reducing heart rate and inhibiting renin release. Diuretics, on the contrary, promote sodium and water excretion, leading to a reduction in plasma volume and cardiac output, and consequently a decrease in blood pressure. However, this reduction in volume stimulates renin release from the kidneys, activating the renin–angiotensin–aldosterone system and initiating compensatory mechanisms aimed at restoring blood pressure. Surprisingly, current treatments seem to entirely disregard the venous district. For example, a recent review on hypertensive drugs (Laurent, 2017) does not even mention the venous system, except to note that certain drugs do not affect it.

The venous district has been shown to manifest significant differences in normotensives compared to hypertensive humans. In particular, a series of experiments in the 1970s showed that there is a substantial reduction in total cardiovascular compliance in hypertensive patients compared to normotensive subjects (London, Safar, Weiss, et al., 1978; London, Safar, Simon, et al., 1978; Safar et al., 1979; London et al., 1982; Safar & London, 1985, 1987). Given that the contribution of the arterial system to total vascular compliance is minimal, these results imply that the venous district is altered in hypertensives. These observations were also corroborated in spontaneously hypertensive rats (Samar & Coleman, 1979), in which venoconstriction emerges at early (developmental) stages of the disease (Martin et al., 1998). More recently similar direct observations on the mesenteric venous system of spontaneously hypertensive rats were provided (Emans et al., 2024), suggesting anomalous sympathetic activity as a possible mechanism for reduced venous compliance in hypertension.

Mathematical models of the cardiovascular system have been used in the past to characterize the role played by different elements of this system in the determination of systemic arterial blood pressure. Cavani et al. (2001) published a modelling study revealing the importance of venous capacitance in dialysis‐induced changes in blood pressure, determining a major role. Furthermore, Yu et al. (2024) studied the role of venous capacity in fluid retention, highlighting that this component had a substantial impact. Finally, some of the authors of this study performed an extensive sensitivity analysis on an anatomically detailed arterio‐venous model Müller et al. (2023), which revealed how the venous system properties are among the most relevant aspects of the cardiovascular system in determining systemic arterial pressure.

In this work, we adopt a mathematical model for the entire cardiovascular system (Müller & Toro, 2014; Toro et al., 2022) that was parametrized in Celant et al. (2023) to represent adaptations of the cardiovascular system observed in hypertensive patients. We translated such adaptations into model parameter modifications and validated the resulting model state, which was shown to satisfactorily represent the hypertensive state. In this modelling set‐up, we considered five major compartments of the cardiovascular system, namely the heart, the systemic arteries, the pulmonary circulation, peripheral beds and the systemic venous system, and quantified the impact of adaptation in each compartment on the determination of the hypertensive state.

In our study, we considered uncertain adaptation parameters by introducing uniformly distributed multiplicative coefficients for adaptations in each compartment. The resulting stochastic model outputs allowed us to assess the effects of individual model adaptations on brachial pressure and, thus, on variables used to diagnose hypertension. Our results show that the role played by changes in the venous system is as relevant as that played by the increment of peripheral resistance. These findings indicate that the venous system is largely responsible for the onset and development of essential hypertension. Therefore, the venous system should be considered a potential treatment target for research on hypertension treatment strategies.

## Methods

### Computational model

**Figure 1:**
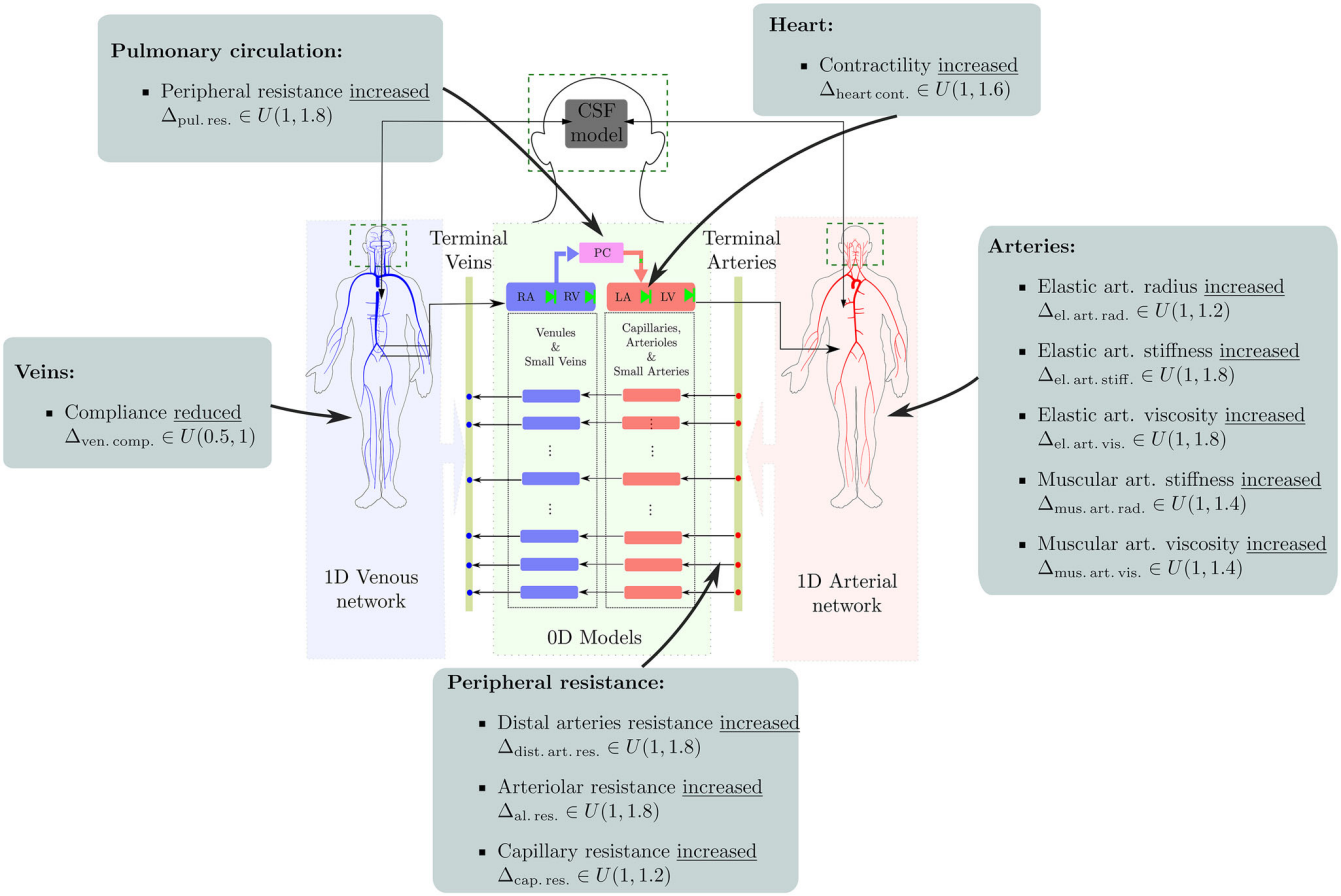
Schematic representation of the closed‐loop model used in this study Grey boxes identify five cardiovascular compartments and show the way hypertension was modelled by introducing changes to the parametrization used in the normotensive case. Perturbations $\Delta_{\mathrm{comp}}$ are computed as specified in eqn (2).

We adopted the version of our global model reported in Celant et al. (2023) without active control mechanisms. The model represents the circulation as a closed loop including systemic and pulmonary circulation, together with a four‐chamber time‐varying elastance heart model. Blood flow in systemic arteries and veins is described by a one‐dimensional wave propagation model, while peripheral and pulmonary circulations are represented by means of lumped parameter models. The mathematical model was numerically solved using a second‐order finite volume local time stepping numerical method (Müller et al., 2016) for non‐linear hyperbolic PDE systems; see also Toro (2001, 2009). Furthermore, we used the explicit Euler method to discretize ordinary differential equations. Deterministic simulations were run for 160 s to ensure that a periodic solution was reached. Figure 1 provides a schematic representation of the model. Figure 1, along with Table 1, provides an overview of how we modelled hypertension.

**Table 1:** Characterization of the hypertensive scenario

Cardiovascular compartment	Parameter/s	Average perturbation	Distribution	Eq. number in Celant et al. (2023)
Heart	Active elastance – $E_{A}$	$\delta_{\mathrm{heart}\;\mathrm{cont}.}=0.3$	$\gamma_{\mathrm{heart}}\in U\left(0,2\right)$	(A9)
	Passive elastance ‐ $E_{B}$			(A9)
Pulmonary circulation	Arterial resistance – $R_{A}$	$\delta_{\mathrm{pul}.\;\mathrm{res}.}=0.4$	$\gamma_{\mathrm{pul}}\in U\left(0,2\right)$	(A14)
Elastic arteries	Radius – $r_{0}$	$\delta_{\mathrm{el}.\;\mathrm{art}.\;\mathrm{rad}.}=0.1$	$\gamma_{\mathrm{art}}\in U\left(0,2\right)$	(A2)
	Pulse wave velocity – $c_{0}$	$\delta_{\mathrm{el}.\;\mathrm{art}.\;\mathrm{stiff}.}=0.4$	$\gamma_{\mathrm{art}}\in U\left(0,2\right)$	See Celant et al. (2023)
	Viscoelasticity – Γ	$\delta_{\mathrm{el}.\;\mathrm{art}.\;\mathrm{vis}.}=0.4$	$\gamma_{\mathrm{art}}\in U\left(0,2\right)$	(A2)
Muscular arteries	Pulse wave velocity – $c_{0}$	$\delta_{\mathrm{musc}.\;\mathrm{art}.\;\mathrm{stiff}.}=0.2$	$\gamma_{\mathrm{art}}\in U\left(0,2\right)$	See Celant et al. (2023)
	Viscoelasticity – Γ	$\delta_{\mathrm{musc}.\;\mathrm{art}.\;\mathrm{vis}.}=0.2$	$\gamma_{\mathrm{art}}\in U\left(0,2\right)$	(A2)
Small arteries,	Arterial impedance – $R_{da}$	$\delta_{\mathrm{dist}.\;\mathrm{art}.\;\mathrm{res}.}=0.4$	$\gamma_{\mathrm{res}}\in U\left(0,2\right)$	(A6)
arterioles and capillaries	Arterioles resistance ‐ $R_{al}$	$\delta_{\mathrm{al}.\;\mathrm{res}.}=0.4$	$\gamma_{\mathrm{res}}\in U\left(0,2\right)$	(A6)
	Capillaries resistance – $R_{cp}$	$\delta_{\mathrm{cap}.\;\mathrm{res}.}=0.1$	$\gamma_{\mathrm{res}}\in U\left(0,2\right)$	(A6)
Venous circulation	Venous compliance – $C_{\mathrm{ven}}$	$\delta_{\mathrm{ven}.\;\mathrm{comp}.}=-0.25$	$\gamma_{\mathrm{ven}}\in U\left(0,2\right)$	See Celant et al. (2023)

Perturbations δ and uniform distributions γ are applied as specified in eqn (1). The last column shows the number of the equation in which the parameter appears in the original publication Celant et al. (2023). For pulse wave velocity $c_{0}$ and venous compliance $C_{\mathrm{ven}}$ the reader is referred to Celant et al. (2023) for the precise use of the parameter in the overall model parametrization.

### Modelling hypertension

The adaptation of the cardiovascular system to hypertension is modelled as in Celant et al. (2023). In short, we modify model parameters to account for differences in mechanical and geometrical properties of the cardiovascular system observed between normo‐ and hypertensive subjects, including changes due to ageing. In particular, we modify heart contractility, pulmonary peripheral resistance, stiffness and geometry of systemic arteries, systemic peripheral resistance and venous compliance of our normotensive patient to reproduce the state of a hypertensive middle‐aged subject (50–65 years old). A detailed explanation of each of these modifications and a discussion on the determination of specific alteration entities can be found in Celant et al. (2023). Here we provide a brief description of modifications made to our normotensive model to represent a hypertensive state:

- Heart contractility: Elevated systemic load induced by hypertension results in left ventricular hypertrophy. The enlargement and thickening of the left ventricular heart muscle help maintain normal systolic wall stress despite the increased load. Active and passive elastances were increased by 30%, as in Segers et al. (2001), to normalize left ventricle wall stress to a normotensive value.
- Systemic arteries: Hypertension‐induced arterial stiffening due to remodelling of vessel walls is addressed as follows. In elastic arteries, we increase the stiffness of arteries so that the wave propagation velocity increases by 40% in elastic arteries and by 20% in muscular arteries to account for differentiated remodelling in these two types of arteries (Laurent & Boutouyrie, 2015; Feihl et al., 2009). Furthermore, we account for the diameter enlargement of elastic arteries due to ageing by introducing a 10% increment, as reported in Bussy et al. (2000). Moreover, we increased the viscous properties of the arterial vessel walls by the same amount used to increase the arterial stiffness parameter, as this parameter is proportional to changes in wall thickness.
- Systemic peripheral resistance: The main site for structural resistance elevation is the proximal part of the microcirculation. For this reason we increase proximal arterial resistance (represented in our model by proximal resistances $R_{da}$ and arteriolar resistances $R_{al}$) by 40% to account for a decrease in small vessel radii of about 8% and an increase in blood viscosity, which ultimately impacts the effective resistance of peripheral beds (Levy et al., 2001; Laurent & Boutouyrie, 2015; Mayet, 2003; Rizzoni & Agabiti‐Rosei, 2012). Resistance vessels ensure that the mean intra‐capillary pressure remains within a tightly controlled range well below the arterial level, preserving the structural integrity of the fragile capillary wall. Therefore, capillary resistance $R_{cp}$ increases by only 10% to account for the reduction of capillary density that characterizes the ageing process (Levy et al., 2001; James et al., 2006).
- Pulmonary peripheral resistance: In hypertension, pulmonary arterial pressure and resistance increase similar to what occurs in the systemic circulation (Lund‐Johansen, 1980; Itelman et al., 2021). Consequently, pulmonary peripheral resistance was increased by 40%, matching the adjustment used for systemic resistance. This allowed us to achieve an increment in pulmonary arterial pressure comparable to that reported by Ferlinz (1980), while assuming minimal variations in cardiac output (Ganau et al., 1992). No changes in pulmonary compliance were incorporated into our hypertensive model parametrization, as studies have shown that intrathoracic vascular compliance remains comparable between normotensive and hypertensive patients (London et al., 1982; Safar et al., 1987).
- Venous system: The significantly larger contribution of venous system compliance to total vascular compliance is a well‐established fact (Safar & London, 1987). Furthermore, human and animal studies have shown that total vascular compliance is reduced in hypertension (London, Safar, Weiss, et al., 1978; London, Safar, Simon, et al., 1978; Safar et al., 1979; London et al., 1982; Safar & London, 1985, 1987; London et al., 1996). According to what was reported in these works, we reduced systemic venous compliance by 25% in our hypertensive model.
- Total blood volume: Total blood volume in essential hypertensive patients is similar to that of normotensive subjects (Ulrych et al., 1969; London et al., 1982; Widgren et al., 1992). Therefore, we kept the total blood volume constant in our hypertensive model.

### Stochastic model

Whereas in Celant et al. (2023) we considered adaptations of the cardiovascular system to ageing and hypertension as deterministic quantities derived from clinical literature, in this work we regarded them as random variables. This was done to evaluate the effect of uncertainty in the chosen hypertensive parametrization on the relevance of considered adaptations. Briefly random parameters were defined as

(1) $$\rho_{\mathrm{ht}}=\rho_{\mathrm{nt}}\;\Delta_{\rho }\;,\;\rho \in P\;,$$

with $\Delta_{\rho }$ a uniformly distributed random variable given by

(2) $$\Delta_{\rho }=\left(1+\delta_{\rho }\gamma_{c}\right)$$

and P={heart.cont.,pul.res.,el.art.rad.,el.art.stiff.,el.art.vis.,musc.art.stiff.,musc.art.vis.,

dist.art.res.,al.res.,cap.res.,ven.comp.}. Furthermore: heart.cont.: heart contractility; pul.res.: pulmonary arterial resistance; el.art.: elastic arteries reference radius; el.art.stiff.: elastic arteries stiffness; el.art.vis.: elastic arteries viscosity; musc.art.stiff.: muscular arteries stiffness; musc.art.vis.: muscular arteries viscosity; dist.art.res.,: distal arteries resistance; al.res.: arteriolar resistance; cap.res.: capillary resistance; ven.comp.: venous compliance. Perturbation $\delta_{\rho }$ is a constant, such that $1+\delta_{\rho }$ is the modified variable used in Celant et al. (2023), and $\gamma_{c}\in U\left(0,2\right)$, so that $\Delta_{\rho }$ is a uniformly distributed random variable with average $1+\delta_{p}$ that varies between the normotensive parameter value and a value with a perturbation twice as high as the one used in Celant et al. (2023). In this way our stochastic model accounts for uncertainty in how hypertension is parameterized, exploring scenarios spanning two extremes: (a) a parameter is left unchanged; (b) the literature‐based variation is only half of the true variation. If, across this range, the sensitivity of arterial pressure does not change substantially, then it is reasonable to assume that we are not missing any key effects with respect to the considered parameters.

We considered c∈C={Heart,Pul.Circ.,Art.,Res.,Veins}, so that the considered compartments are: heart, pulmonary circulation, systemic arteries, peripheral arteries (resistance vessels) and venous system. Values for $\delta_{\rho }$ and $\gamma_{\mathrm{comp}}$ are reported in Table 1. This implies that we have introduced five stochastic variables to characterize the variability and uncertainty of different adaptation types. Consequently, the model incorporates stochastic parameters and becomes inherently stochastic. To discretize the problem and analyse the impact of parameter variability on the results, we used polynomial chaos with stochastic collocation, as described by Xiu (2010). We ensured that the refinement level of the nested quadrature rule was sufficient to yield mesh‐independent statistical moments of the variables of interest. In particular, we used the open‐source library Chaospy (Feinberg & Langtangen, 2015) to generate a polynomial approximation of the variables of interest over the parameter space. We employed a nested quadrature rule and applied it for four levels, as this proved sufficient to obtain level‐independent statistical moments for variables of interest. Level‐independence was defined as a variation in the expected value and the SD of the variables of interest of less than 5% between two successive levels. Considered variables of interest were the ones reported in Table 2.

**Table 2:** Cardiovascular indexes

	Normotensive		Hypertensive			
Index	Model	Ref.	Model	Stoch. Model	Ref.	Ref.
Brachial SBP [mmHg]	110.32	115 ± 11	155.69	156.53 ± 22.51	153 ± 22	Bussy et al. (2000)
Brachial DBP [mmHg]	72.67	71 ± 7	93.06	93.98 ± 11.71	99 ± 10	Bussy et al. (2000)
Brachial MBP [mmHg]	88.81	86 ± 8	120.68	121.98 ± 15.90	119 ± 12	Bussy et al. (2000)
Brachial PP [mmHg]	37.66	38 ± 2.8	62.64	62.56 ± 13.21	56.4 ± 14.7	Li et al. (2020)
Carotid SBP [mmHg]	109.04	118 ± 14	155.99	155.84 ± 21.75	166 ± 24	Bussy et al. (2000)
Carotid DBP [mmHg]	73.17	70 ± 7	93.48	94.98 ± 11.72	95 ± 12	Bussy et al. (2000)
Carotid PP [mmHg]	35.87	48 ± 15	62.51	60.86 ± 10.81	71 ± 24	Bussy et al. (2000)
Aortic SBP [mmHg]	105.56	108 ± 12	154.49	154.00 ± 21.73	140 ± 17	McEniery et al. (2008)
Aortic DBP [mmHg]	73.64	75 ± 8	94.01	95.14 ± 11.64	88 ± 11	McEniery et al. (2008)
Aortic PP [mmHg]	31.92	33 ± 10	60.48	58.85 ± 10.77	52 ± 17	McEniery et al. (2008)
cfPWV [m/s]	7.14	8.5 ± 1.5	11.62	11.25 ± 3.16	11.8 ± 2.7	Asmar et al. (1995)
baPWV [m/s]	8.98	14.84 ± 3.4	13.52	14.74 ± 2.62	16.7 ± 3.6	Tanaka et al. (2009)
C_a_ index [mL/mmHg/m^2^]	0.98	1.08 ± 0.25	0.62	0.64 ± 0.13	0.61 ± 0.19	Abdelhammed et al. (2005)
CI [L/min/m^2^]	2.73	2.9 ± 0.8	2.93	2.94 ± 0.30	3.1 ± 0.8	Ganau et al. (1992)
SI [mL/m^2^]	36.81	43 ± 9	39.09	39.49 ± 3.33	45 ± 11	Ganau et al. (1992)
E_a_ [mmHg/mL]	1.35	1.52 ± 0.1	1.73	1.80 ± 1.02	1.8 ± 0.17	Bonnet et al. (2017)
E_es_ [mmHg/mL]	2.21	2.03 ± 0.2	2.76	2.82 ± 1.45	2.43 ± 0.2	Bonnet et al. (2017)
E_a_/E_es_	0.61	0.77 ± 0.04	0.63	0.64 ± 0.13	0.73 ± 0.06	Bonnet et al. (2017)
LV_max_ [ml]	113.88	150 ± 67	122.14	124.70 ± 14.51		Mynard and Smolich (2015)
LV 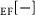	0.62	0.68 ± 0.12	0.61	0.61 ± 0.05		Mynard and Smolich (2015)
RV_max_ [ml]	124.78	173 ± 95	144.50	146.11 ± 12.48		Mynard and Smolich (2015)
RV 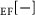	0.56	0.57 ± 0.10	0.52	0.52 ± 0.02	0.59 ± 0.07	Mynard and Smolich (2015) Ferlinz (1980)
RV‐SP [mmHg]	24.72	22 ± 6	33.07	33.57 ± 3.93	27 ± 5	Ferlinz (1980)
RV‐EDP [mmHg]	5.43	3 ± 2	6.52	6.62 ± 0.72	5 ± 2	Ferlinz (1980)
MPAP [mmHg]	15.74	12 ± 3	20.98	21.29 ± 2.29	17 ± 5	Ferlinz (1980)
SPAP [mmHg]	24.11	20 ± 6	32.41	32.87 ± 3.83	26 ± 6	Ferlinz (1980)
DPAP [mmHg]	10.39	8 ± 2	13.59	13.81 ± 1.43	11 ± 5	Ferlinz (1980)
MPVP [mmHg]	8.78	—	12.05	12.28 ± 1.55	—	—
MCVP [mmHg]	4.06	4.4 ± 0.6	4.93	5.01 ± 0.59	5.0 ± 0.6	Safar et al. (1979)

Model: (S/D)BP: systolic/diastolic blood pressure; MBP: mean blood pressure; PP: pulse pressure; cfPWV: carotid‐femoral pulse wave velocity; baPWV: brachial‐ankle pulse wave velocity; Augmented P: augmented pressure; C_a_ index: total arterial compliance index (Abdelhammed et al., 2005); CI: cardiac index; $E_{a}$: arterial elastance; $E_{es}$: left ventricle elastance; (L/R)V: left/right ventricle volume; EDP: end‐diastolic pressure; PAP: pulmonary artery pressure; M(P/C)VP: mean pulmonary/central venous pressure. Details on how to compute these indexes can be found in Appendix.

## Results

**Figure 2:**
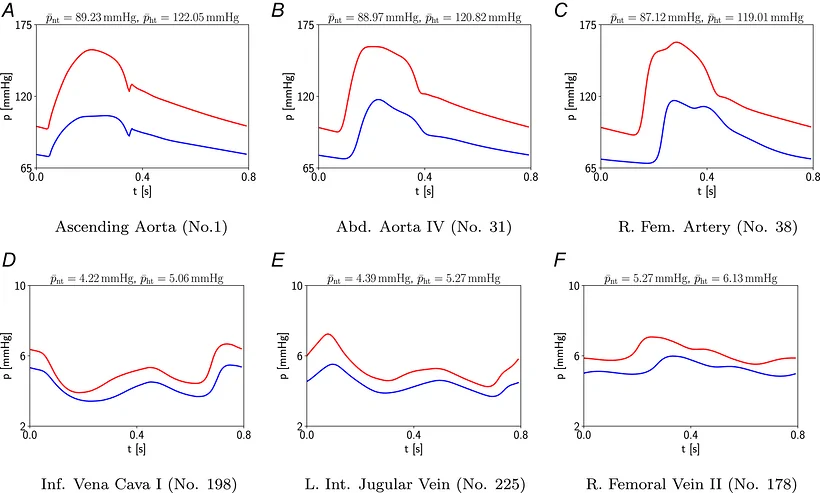
Predicted blood pressure waveforms Computed blood pressure at selected arterial and venous vessels in normotensive (blue line) and hypertensive (red line) states. Cardiac cycle averaged values are denoted by ${\bar{p}}_{\mathrm{nt}}$ (normotensive) and ${\bar{p}}_{\mathrm{ht}}$ (hypertensive).

Table 2 reports cardiovascular indexes obtained from using our normo‐ and hypertensive model parametrizations, along with reference values from the literature. For the hypertensive case we report results for both the deterministic model, where we set parameter perturbations to $p^{\mathrm{ht}}=p^{\mathrm{nt}}\;\left(1+\delta_{p}\right)$, and the stochastic model, with random parameter perturbations defined in eqns (1) and (2). In the latter case, we report the expected value ± SD of computed output variables. A clarification regarding SD is in order. Whereas the SD in results obtained with the stochastic model reflects the impact of uncertain model input parameters on computed indexes, the SD reported for literature data indicates the variability of indexes in the population for which they were computed. Therefore, we expect these two quantities to be unrelated. In order to further characterize the normo‐ and hypertensive states, Fig. 2 displays pressure waveforms for selected arteries and veins in the normo‐ and hypertensive cases.

**Figure 3:**
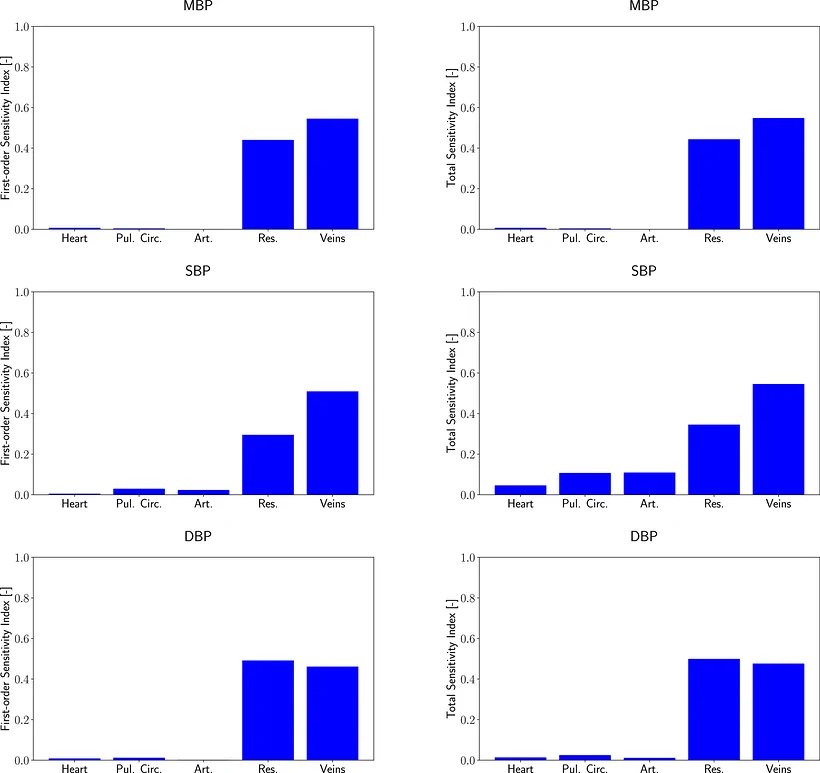
Global sensitivity analysis First‐order (left column) and total (right column) sensitivity indexes for mean (MBP), systolic (SBP) and diastolic (DBP) brachial pressure.

The sensitivity of predicted outputs to uncertain parameters can be studied in Fig. 3, which shows first‐order and total sensitivity indexes for mean, systolic and diastolic brachial pressure. First‐order sensitivity indexes show the contribution of each random parameter to the variance of a variable of interest. In contrast, total sensitivity indexes show the impact of a random parameter's variability plus the effects resulting from its interactions (of any order) with other random parameters.

**Figure 4:**
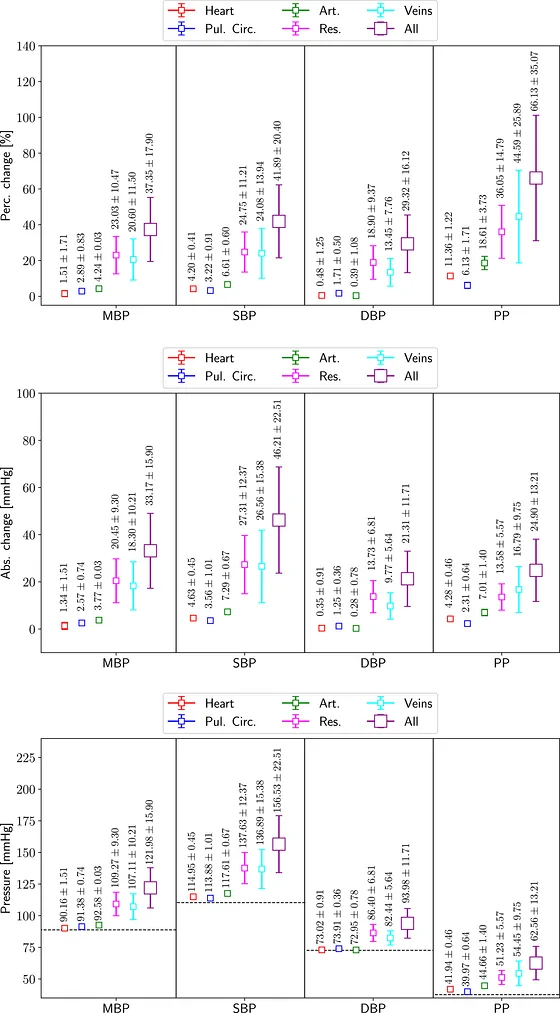
Impact of parameter changes on systemic arterial pressure Square boxes represent mean values, whereas error bars denote the SD. Dashed horizontal lines represent the normotensive model output values. MBP: mean blood pressure; SBP: systolic blood pressure; DBP: diastolic blood pressure; PP: pulse pressure. All values are computed using pressure sampled at the midpoint of vessel 17 in Toro et al. (2022), corresponding to the left brachial artery.

**Figure 5:**
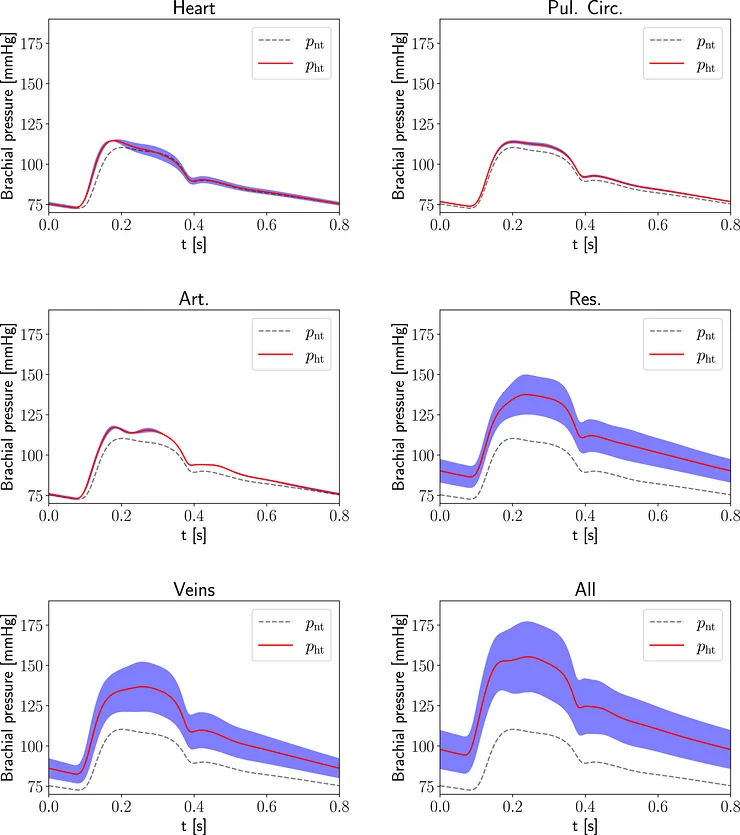
Brachial artery pressure waveforms The normotensive case's results are shown in a dashed grey line. In contrast, the continuous red line displays the hypertensive case stochastic results, with a shaded area representing limits for ±1 SD. Results are shown for one‐at‐a‐time compartment parameter changes and for the case in which all compartments are affected, with sub‐plot titles indicating the compartment in which parameters were altered.

Figure 4 illustrates changes in mean, systolic and diastolic and pulse brachial pressure with respect to normotensive values, obtained when considering the adaptation of the different cardiovascular system compartments to hypertension in a one‐at‐a‐time fashion. Changes are reported in three ways: percentage and absolute changes, as well as absolute pressure values. Moreover, Fig. 5 displays the brachial artery pressure waveforms for all these cases, along with those obtained with the deterministic normotensive model.

## Discussion

Our results clearly highlight the major role played by the venous system in the determination of the hypertensive state. In fact, the contribution of reduced venous compliance to elevated arterial pressure is second only to the increment observed in peripheral resistance. In this section we first discuss the validity of the predicted normo‐ and hypertensive states, and then delve into a discussion of major determinants of hypertension, from which the prominent role of the venous system emerges. Motivated by this fact, we direct our attention to the venous system in hypertension.

### Characterization of normo‐ and hypertensive states

Most indexes reported in Table 2 compare satisfactorily to reference values for normo‐ and hypertensive subjects. Noteworthy, we observe that this is true for indexes that vary significantly between the normo‐ and the hypertensive states, namely systolic, diastolic and mean pressure (SBP, DBP and MBP), for all observed arteries, carotid‐femoral pulse wave velocity (cfPWV), total arterial compliance index ($C_{a}$), arterial and left ventricle elastances ($E_{a}$ and $E_{es}$), right ventricle systolic pressure (RV‐SP) and mean, systolic and diastolic pulmonary pressures (MPAP, SPAP and DPAP). In addition we see that indexes that are known to be similar for normo‐ and hypertensive subjects follow this pattern also in our results; see, for example, the cardiac index (CI), the stroke index (SI), the ratio between arterial and left ventricle elastances ($E_{a}/E_{es}$) and the right ventricle ejection fraction (${\text{RV}}_{\text{EF}}$). Remarkably, another variable that is not largely altered by hypertension, at least in absolute terms, is the mean central venous pressure (CVP). In fact, our results show a 1 mmHg increment in CVP when moving from the normotensive to the hypertensive scenario, in line with experimental observations (Safar et al., 1979). The reader is referred to Celant et al. (2023) for an in‐depth discussion on the physiological validity of the characterization of normo‐ and hypertensive states by our model.

### Major determinants of hypertension

Table 2 allowed us to study the effect of uncertainty in hypertension modelling on computed indexes. In fact, parameters are assumed to vary between the normotensive and twice its hypertensive value. This variability translates into significant SD values of computed haemodynamic indexes. For example, the SDs of computed systolic and diastolic brachial pressures are 22.51 and 11.71 mmHg, respectively. At this point, it becomes interesting to understand the contribution of each altered compartment's uncertainty to the overall uncertainty observed in output variables. This is achieved through a variance‐based sensitivity analysis, where computed sensitivity indexes are based on the stochastic nature of model outputs when random input parameters are considered (Saltelli et al., 2008). Sensitivity indexes displayed in Fig. 3 show that the increment of peripheral resistance and the reduction in venous compliance are the two most important adaptations in determining MBP, DBP and SBP variability. Uncertainty in all other considered compartments has little impact on the final uncertainty shown by variables of interest.

The dominant role of peripheral resistance and venous compliance changes in determining stochastic output variability suggested that these two quantities could play a privileged role in causing the hypertensive state. To explore this aspect, we performed stochastic simulations in which adaptation to hypertension was modelled in a one‐at‐a‐time fashion, while keeping unchanged normotensive parameter values in all other compartments. The resulting changes in mean, systolic, diastolic and pulse brachial pressure (with respect to normotensive values) are reported in Fig. 4. Our results show that modifications in peripheral resistance and venous compliance have significantly greater impact than those in any other compartment. Moreover, these results demonstrate that even substantial parameter uncertainty related to adaptations in the heart, pulmonary circulation and systemic arteries does not lead to significant increments in brachial pressure indexes. Figure 5 displays brachial artery pressure waveforms obtained with the deterministic normotensive model, with the stochastic hypertensive model for cases in which a one‐at‐a‐time parameter modification was introduced, and for the case in which all parameters are modified to represent the hypertensive state. We see that adaptations of systemic arteries and the heart manifest as uncertainty in brachial pressure at different parts of the cardiac cycle. Heart adaptations concentrate uncertainty in brachial artery pressure during late systole and initial diastole, whereas changes in the arterial system result in more considerable uncertainty around peak systole. On the contrary, adaptations of the pulmonary circulation, peripheral systemic resistances and the venous system cause uncertainty in brachial pressure that is uniformly distributed over the cardiac cycle, with significantly more uncertainty for adaptations of peripheral systemic resistances and the venous system.

Clearly, our results depend strongly on how we characterized the hypertensive state. This was a two‐step process where we first identified deterministic parameter changes based on the clinical literature, $1+\delta_{\rho }$ in eqn (2), and then accounted for uncertainty in the parametrization of the hypertensive state by assuming that altered parameters were stochastic, uniformly distributed between the normotensive default value and twice the literature‐reported alteration. We showed that, under this uncertainty model, changes in the venous system consistently remained a highly relevant alteration, together with peripheral resistance. Finally, it is worth noting that although the stochastic approach allowed us to assess the impact of potential errors on the determination of deterministic parameters over a wide range of uncertainty, all results provided here should be considered as strongly dependent on our modelling assumptions, and their validity is limited to uncertainty within explored ranges.

### The venous system in hypertension

The prominent role of the venous system as a key determinant of hypertension emerges clearly from our results. This finding motivates a more detailed discussion of the venous system's involvement in hypertension. In what follows, we first review previous work on how venous properties affect arterial pressure, and then focus on two main aspects: reduced venous compliance and elevated venous pressure.

#### Venous system properties as a major determinant of arterial pressure

The dominant role played by venous system alterations in the determination of the hypertensive state is connected to the intrinsic sensitivity of arterial pressure to venous‐related parameters. In fact, several modelling studies based on closed‐loop cardiovascular models have shown that the venous system is one of the major determinants of systemic arterial pressure across diverse physiological and clinical contexts, including healthy normotensive young adults Müller et al. (2023), haemodialysis‐induced hypovolaemia Cavani et al. (2001) and fluid retention in chronic kidney disease Yu et al. (2024). These results indicate that venous alterations contribute to hypertension not only because of the magnitude of such alterations but also because arterial pressure is intrinsically sensitive to venous properties.

#### Reduced venous compliance

Alteration in the venous system compliance, indirectly measured in hypertensive humans, was observed in multiple early studies (London, Safar, Weiss, et al., 1978; London, Safar, Simon, et al., 1978; Safar et al., 1979; London et al., 1982; Safar & London, 1985, 1987; London et al., 1996). These results were also found in spontaneously hypertensive rats Samar and Coleman (1979) and reproduced recently in (Emans et al., 2024). In the latter work, reduced venous compliance was associated with elevated sympathetic venoconstriction. There is substantial research surrounding sympathetic induced venoconstriction in hypertension Fink (2009) and its critical role during the early stages of hypertension. Interestingly, borderline hypertension subjects showed reduced venous compliance in Takeshita and Mark (1979), a finding that was also determined experimentally in animal models Martin et al. (1998). Furthermore, in normotensive subjects with a family history of hypertension, venous compliance was reduced (Ito et al., 1986; Widgren et al., 1992).

In our work, we modelled experimentally observed changes in total effective vascular compliance as a reduction of systemic venous compliance. Experimental observations regard an effective parameter, that is, a parameter that reflects the coordinated response of multiple components of the circulation to stimuli. The processes and physical components of the cardiovascular system that contribute to this response are multiple. For example, increased venous tone can have a neural component and a structural one. By contrast, in our model, systemic venous compliance is a well‐defined mechanical property. Although we could have decided to act on the unstressed venous volume, we decided to control venous compliance, as its link to total effective compliance is physically closer than that of unstressed venous volume. Furthermore, in a previous work Celant et al. (2021), we showed that this approach results in similar changes in total effective vascular compliance measured in hypertensive patients compared with that observed in normotensive ones London, Safar, Weiss, et al. (1978).

#### Is venous pressure increased in hypertension?

Caliva et al. (1963) showed that the mean finger venous pressure in the normotensive subjects was 8.5±3.1 mmHg, compared with 10.9±3.1 mmHg in the hypertensive patients (P<0.01). Moreover, they observed that 90% of the hypertensive subjects had a venous pressure between 8.9 and 13.8 mmHg, whereas 90% of the normotensive ones fell between 5.9 and 10.4 mmHg. They concluded that *there is a small but definite increase in digital venous pressure in the hypertensive state*.

More recently Novo et al. (1997), using a Doppler ultrasound instrument, found that venous pressure was 14.76±1.90 mmHg in one hypertensive group, versus 12.53±2.39 mmHg in another hypertensive group, versus 8.7±2.02 mmHg in the control group (P<0.0001). The authors concluded *that hypertension is a disease in which both the arterial and the venous vascular beds are involved with increased pressure in both circulatory beds*.

London, Safar, Weiss, et al. (1978) reported measured central venous pressure values of 4.5±0.6 mmHg in normotensives and 5.5±0.6 mmHg in hypertensives, with an increase regarded as not statistically significant. Similar results are found in London, Safar, Weiss, et al. (1978); they reported measured central venous pressure as 4.2±0.8 mmHg in normotensives and 5.7±0.4 mmHg in hypertensives, and the increase was again regarded non‐significant.

Notably, statistical significance tests are highly dependent on the SD of the sampled data. In addition, the accuracy by which venous pressure can be measured is certainly much lower than that of arterial pressure measurements. These two factors combined may contribute to the lack of statistically significant differences by several studies between normotensive and hypertensive venous pressures.

Our present computational study predicts a rise in venous pressure of about 25%, as reported in Table 2 and also seen in Fig. 2. Contrary to experimental results, our model predictions are not affected by measurement errors, and a small, in absolute terms, but 25% in relative terms, pressure increment in the hypertensive state is related to highly perturbed arterial pressure.

Although the evidence for increased venous pressure in arterial hypertension is currently not sufficiently strong, it certainly warrants further studies. However, if venous pressure were elevated in arterial hypertension, one could speculate that the increased post‐capillary pressure might disturb interstitial fluid reabsorption into the circulation. Reduced plasma volume, measured in hypertensives by London, Safar, Weiss, et al. (1978), may naturally be associated with increased haematocrit. Moreover, several studies have indicated that haematocrit is related to the risk of hypertension. For example, Jae et al. (2014) suggested that higher haematocrit in men predicts the development of hypertension over time. Paul et al. (2012) demonstrated that increased haematocrit predicts long‐term mortality in hypertensive adults. Piesanen et al. (2023) associated increased haematocrit to higher hypertension risk in normotensive young subjects. The precise mechanisms by which elevated haematocrit would explain increased blood pressure in hypertensives warrants further analysis, which lies outside the scope of the present paper. However, as would be expected, there is evidence Cinar et al. (1999) that increased haematocrit is associated with increased whole blood viscosity. This, via Poiseuille's law, would result in increased vascular resistance, a well‐established factor in hypertension.

The current model indicates that isolated modulation of venous compliance exerts direct causal effects on arterial pressure, contributing to the development of hypertension; this supports the hypothesis that reduced venous compliance can initiate and sustain a hypertensive state. The effect of existing therapeutic strategies to ‘inadvertently’ change venous compliance and where these changes fit within the sequence of hypertensive progression warrants further investigation.

## Additional information

## Competing interests

The authors declare that they have no competing interests that could appear to influence the work reported in this paper.

## Author contributions

E.T. and L.M. conceptualised the study; acquired, analysed, and interpreted the data; drafted and critically revised the work; approved the final version; and agreed to be accountable for all aspects of the work. T.E., F.M., and J.P. acquired, analysed, and interpreted the data; drafted and critically revised the work; approved the final version; and agreed to be accountable for all aspects of the work.

## Funding

The study was funded by EC | NextGenerationEU (NGEU) under grant number CN_00000013 awarded to Lucas Omar Müller.

## Generative AI statement

Generative AI tools were used only for language editing and stylistic improvement. No AI tool was used to generate scientific content, analyse data, create figures, or draw conclusions. The authors reviewed and approved the final manuscript and take full responsibility for its content.

## Supporting information


Peer Review History

## Data Availability

This study is based solely on mathematical modelling and simulation. No experimental or observational data were generated or analysed. Therefore there is no associated dataset.

## Indexes

In this appendix we summarize the main cardiovascular indexes adopted in this work and how they are defined and calculated, in particular those in Table 2. All the indexes were evaluated from the numerical solution in the last cardiac cycle of a simulation that had reached a periodic solution. Therefore, in the following list, we refer to maximum, minimum and mean values over a cardiac cycle. For one‐dimensional vessels computed values are extracted from pressure, flow or velocity waveforms evaluated at the middle‐point of the vessel's length.

- SBP: systolic blood pressure; maximum blood pressure.
- DBP: diastolic blood pressure; minimum blood pressure.
- MBP: mean blood pressure; average blood pressure.
- PP: pulse pressure; the difference between maximum and minimum blood pressures.
- cfPWV: carotid‐femoral pulse wave velocity; it is evaluated with the ‘foot‐to‐foot’ method as
  $$\text{cf}\text{PWV}=\frac{\Delta L}{\Delta t}\;,$$
  where Δt is the time delay between the arrival of pulse at the right common carotid artery and at the right femoral artery and ΔL is the distance between the two measurement points.
- baPWV: brachial‐ankle pulse wave velocity; it is evaluated with the ‘foot‐to‐foot’ method as
  $$\text{baPWV}=\frac{\Delta L}{\Delta t}\;,$$
  where Δt is the time interval between the arrival of pulse at the right brachial artery and at the right tibial artery and ΔL is the distance between the two measurement points.
- Augmented P: augmented pressure; it is calculated as the difference between the second and first systolic peaks of aortic pressure.
- Augmentation index; it is calculated as the ratio between augmentation pressure and pulse pressure of aortic root.
- C_a_ index: total arterial compliance index; arterial compliance index is evaluated as the ratio between stroke volume and brachial pulse pressure, divided by body surface area, taken as 1.92 $m^{2}$ for both normotensive and hypertensive subjects.
- HR: heart rate.
- CI: cardiac index; it is calculated as the cardiac output divided by body surface area, taken as 1.92 $m^{2}$ for both normotensive and hypertensive subjects.
- SI: stroke index; it is evaluated as stroke volume (end‐diastolic volume – end‐systolic volume of left ventricle) divided by body surface area, taken as 1.92 $m^{2}$ for both normotensive and hypertensive subjects.
- E_a_: arterial elastance; it is calculated as the ratio between left ventricle end‐systolic pressure and stroke volume.
- E_es_: left ventricle elastance; it is evaluated as left ventricle end‐systolic pressure divided by left ventricle end‐systolic volume.
- E_a_/E_es_: arterial‐ventricular coupling index.
- LV/RV_max_: maximum volume of left/right ventricle.
- LV/RV_EF_: ejection fraction of left/right ventricle; it is calculated as the ratio between stroke volume and end‐diastolic volume.
- max/min $\frac{dP}{dt}$: maximum/minimum pressure rate.
- RV‐SP: right ventricle systolic pressure.
- RV‐EDP: right ventricle end‐diastolic pressure.
- M/S/D PAP: mean/systolic/diastolic pulmonary artery pressure, evaluated in the zero‐dimensional compartment of the arteries in the pulmonary circulation.
