# Supplementary material for: Unveiling the role of the venous system in arterial hypertension: A computational study
Source: J Physiol. 2026 Jul 31;604(17):7145–60. doi: 10.1113/JP289895 (PMC13532949; doi:10.1113/JP289895)
Supplement: Supplementary file 1 [file TJP-604-7145-s001.pdf]

The Journal of Physiology

Unveiling the role of the venous system in arterial hypertension: A computational study

Eleuterio F. Toro, Lucas Omar Müller, Tonja W Emans, Fiona D McBryde, and Julian F. R. Paton
DOI: 10.1113/JP289895

Corresponding author(s): Lucas Müller (lucas.muller@unitn.it)

The referees have opted to remain anonymous.

Review Timeline:

Submission Date:	08-Aug-2025
Editorial Decision:	03-Oct-2025
Revision Received:	05-May-2026
Accepted:	03-Jun-2026

Senior Editor: Vaughan Macefield

Reviewing Editor: Mary Margot Maleckar

Transaction Report:

(Note: With the exception of the correction of typographical or spelling errors that could be a source of ambiguity, letters and reports are not edited. Depending on transfer agreements, referee reports obtained elsewhere may or may not be included in this compilation. Referee reports are anonymous unless the Referee chooses to sign their reports.)

Dear Dr Müller,

Re: JP-RP-2025-289895 "Unveiling the role of the venous system in arterial hypertension: A computational study" by Eleuterio F. Toro, Tonja W Emans, Fiona D McBryde, Julian F. R. Paton, and Lucas Omar Müller

Thank you for submitting your manuscript to The Journal of Physiology. It has been assessed by a Reviewing Editor and by 2 expert referees and we are pleased to tell you that it is potentially acceptable for publication following satisfactory major revision.

Please advise your co-authors of this decision as soon as possible.

The referee reports are copied at the end of this email.

Please address all the points raised and incorporate all requested revisions or explain in your Response to Referees why a change has not been made. We hope you will find the comments helpful and that you will be able to return your revised manuscript within 9 months. If you require longer than this, please contact journal staff: jp@physoc.org. Please note that this letter does not constitute a guarantee for acceptance of your revised manuscript.

Your revised manuscript should be submitted online using the link in your Author Tasks: Link Not Available. This link is accessible via your account as Corresponding Author; it is not available to your co-authors. If this presents a problem, please contact journal staff (jp@physoc.org). Image files from the previous version are retained on the system. Please ensure you replace or remove any files that are being revised.

If you do not wish to submit a revised version of your manuscript, you must inform our journal staff (jp@physoc.org) or reply to this email to request withdrawal. Please note that a manuscript must be formally withdrawn from the peer review process at one journal before it may be submitted to another journal.

TRANSPARENT PEER REVIEW POLICY: To improve the transparency of its peer review process, The Journal of Physiology publishes online as supporting information the peer review history of all articles accepted for publication. Readers will have access to decision letters, including Editors' comments and referee reports, for each version of the manuscript, as well as any author responses to peer review comments. Referees can decide whether or not they wish to be named on the peer review history document.

ABSTRACT FIGURES: Authors are expected to use The Journal's premium BioRender account to create/redraw their Abstract Figures. Information on how to access this account is here: https://physoc.onlinelibrary.wiley.com/journal/14697793/biorender-access.

This will enable Authors to create and download high-resolution figures. If authors have used the free BioRender service, they can use the instructions provided in the link above to download a high-resolution version suitable for publication.

The link provided should only be used for the purposes of this submission. Authors will be charged for figures created on this account if they are not related to this manuscript submission.

LANGUAGE EDITING AND SUPPORT FOR PUBLICATION: If you would like help with English language editing, or other article preparation support, Wiley Editing Services offers expert help, including English Language Editing, as well as translation, manuscript formatting, and figure formatting at www.wileyauthors.com/eeo/preparation. You can also find resources for Preparing Your Article for general guidance about writing and preparing your manuscript at www.wileyauthors.com/eeo/prepresources.

RIGOUR AND REPRODUCIBILITY: We recommend authors consult The Journal's Rigour and Reproducibility requirements to ensure that their manuscript is suitable for publication: https://physoc.onlinelibrary.wiley.com/pb-assets/hub-assets/physoc/documents/TJP-Rigour-and-Reproducibility-Requirements.pdf

REVISION CHECKLIST:

Check that your Methods section conforms to journal policy: https://jp.msubmit.net/cgi-bin/main.plex?form_type=display_requirements#methods.

Check that data presented conforms to the statistics policy: https://jp.msubmit.net/cgi-bin/main.plex?form_type=display_requirements#statistics.

Upload a full Response to Referees file. To create your 'Response to Referees': copy all the reports, including any comments from the Senior and Reviewing Editors, into a Microsoft Word, or similar, file and respond to each point, using font or background colour to distinguish comments and responses and upload as the required file type.

Please upload two versions of your manuscript text: one with all relevant changes highlighted and one clean version with no

changes tracked. The manuscript file should include all tables and figure legends, but each figure/graph should be uploaded as separate, high-resolution files.

You may also upload:

- 'Potential Cover Art' for consideration as the issue's cover image
- Appropriate Supporting Information (Video, audio or data set: see https://jp.msubmit.net/cgi-bin/main.plex?form_type=display_requirements#supp).

We look forward to receiving your revised submission.

If you have any queries, please reply to this email and we will be pleased to advise.

Yours sincerely,

Vaughan Macefield
Senior Editor
The Journal of Physiology

REQUIRED ITEMS

1)- Author photo and profile. First or joint first authors are asked to provide a short biography (no more than 100 words for one author or 150 words in total for joint first authors) and a portrait photograph. These should be uploaded and clearly labelled together in a Word document with the revised version of the manuscript. See Information for Authors for further details.

2) - Your manuscript must include a complete Additional Information section, including competing interests; funding; author contributions and acknowledgements.

3) - Please upload separate high-quality figure files via the submission form.

4)- Please ensure that any tables are editable and in Word format, and wherever possible, embedded in the article file itself.

5) - Please ensure that the Article File you upload is a Word file.

6) - Papers must comply with the Statistics Policy: https://jp.msubmit.net/cgi-bin/main.plex?form_type=display_requirements#statistics.

In summary:

- If n {less than or equal to} 30, all data points must be plotted in the figure in a way that reveals their range and distribution. A bar graph with data points overlaid, a box and whisker plot or a violin plot (preferably with data points included) are acceptable formats.

- If n > 30, then the entire raw dataset must be made available either as supporting information, or hosted on a not-for-profit repository, e.g. FigShare, with access details provided in the manuscript.

- 'n' clearly defined (e.g. x cells from y slices in z animals) in the Methods. Authors should be mindful of pseudoreplication.

- All relevant 'n' values must be clearly stated in the main text, figures and tables.

- The most appropriate summary statistic (e.g. mean or median and standard deviation) must be used. Standard Error of the Mean (SEM) alone is not permitted.

- Exact p values must be stated. Authors must not use 'greater than' or 'less than'. Exact p values must be stated to three significant figures even when 'no statistical significance' is claimed.

7) - Please include an Abstract Figure file, as well as the Figure Legend text within the main article file. The Abstract Figure is a piece of artwork designed to give readers an immediate understanding of the research and should summarise the main

conclusions. If possible, the image should be easily 'readable' from left to right or top to bottom. It should show the physiological relevance of the manuscript so readers can assess the importance and content of its findings. Abstract Figures should not merely recapitulate other figures in the manuscript. Please try to keep the diagram as simple as possible and without superfluous information that may distract from the main conclusion(s). Abstract Figures must be provided by authors no later than the revised manuscript stage and should be uploaded as a separate file during online submission labelled as File Type 'Abstract Figure'. Please also ensure that you include the figure legend in the main article file. All Abstract Figures should be created using BioRender. Authors should use The Journal's premium BioRender account to export high-resolution images. Details on how to use and access the premium account are included as part of this email.

8) - Please ensure that all figures and tables have a title and legend, and that they have been cited within the main article text.

EDITOR COMMENTS

Senior Editor:

Thank you for submitting your manuscript to The Journal of Physiology. I have now received comments from two independent reviewers, both experts in the field, and a reviewing editor. While all see merit in your study, there are issues that will need to be addressed before we can give your manuscript further editorial consideration. I look forward to receiving your revised manuscript in due course.

Reviewing Editor:

In this study, the authors use a closed-loop computational model to perform a sensitivity analysis, concluding that reduced venous compliance is a key contributor to the hypertensive state, second only to peripheral resistance. The manuscript is well-written and the approach is interesting. However, both reviewers have raised significant concerns that must be thoroughly addressed before the manuscript can be considered further for publication.

The consensus is that the manuscript requires Major Revision. The primary concerns are outlined below:

1. Insufficient context and acknowledgment of prior work.

The failure to adequately cite and discuss the extensive body of existing literature, both computational and experimental, previously identified the venous system as a key player in blood pressure regulation and hypertension needs to be acknowledged and highlighted. As noted by Referee #2, this is not the first study to highlight this role. The authors must perform a comprehensive literature review and rewrite the Introduction and Discussion:

(a) Acknowledge and integrate prior modeling studies that have explored the role of venous capacitance/compliance.

(b) Discuss the significant experimental work in both animal models and humans that supports this concept (several key examples were provided by Referee #2).

(c) Clearly articulate what their specific modeling approach adds to this established field and how their quantitative findings advance our understanding beyond previous work.

(d) Expand on the distinction between venous compliance and venous capacitance, a nuanced but important point raised by Referee #2.

2. Methodological justification and model limitations.

Referee #1 raised several important points regarding the study's methodology that weaken the physiological relevance of the conclusions. The authors should:

(a) Address the critique that the model was inherently biased towards a hypertensive state. Please discuss whether the same conclusions would be reached if the analysis were performed on a model with different initial parameter settings.

(b) Provide a stronger justification for the specific ranges chosen for the random parameter modifications. The authors must discuss how the arbitrary selection of these ranges might have influenced the final conclusions of the sensitivity analysis.

(c) Strengthen the link between the model's structure and the physiological mechanisms presented in the Discussion. The conclusions are derived from a sensitivity analysis, and the discussion should more explicitly reflect this specific approach.

(d) Acknowledge the limitation that the model was not compared against real physiological signals to provide a goodness-of-fit assessment. While performing this analysis may be beyond the scope of a revision, this limitation should be explicitly stated.

3. Focus of the Discussion.

The authors should consider the suggestion from Referee #2 to streamline the Discussion. The final sections on the revised Starling principle and haemorheology, while interesting, detract from the central message of the paper. Deleting or significantly shortening these sections could be considered.

Methods Details:

Perhaps - if revised, the methods require additional justification. The authors should address concerns that the model was biased toward a hypertensive state from the outset, which may limit conclusions drawn from the sensitivity analysis and provide a more thorough justification for the chosen percentage of parameter variation.

REFEREE COMMENTS

Referee #1:

In the study the authors utilized a previously validated model (Celant et al 2023) to model hypertension. In the present study the authors modified the parameters in a random fashion to assess the sensitivity of the model. This analysis of sensitivity allowed the authors to conclude that the reduced venous compliance might contribute significantly to hypertension in addition to the increase of peripheral resistance.

The study is interesting and well-written. However, physiological mechanisms that might be responsible for the modification of venous system in hypertensive patients remain poorly elucidated.

1) One of the major limitations of the study is that the behavior of the model was biased toward a hypertensive situation. Thus, the conclusion of the study is limited to what the analysis of sensitivity has provided in this specific situation, namely the relative importance of the compartments in a situation of simulated hypertension. It is very important to check whether the same conclusions could be reached in different simulated situations and/or random setting of the mean value of parameters. My guess is that, given the structure of the model, conclusions might be the same regardless of the initial setting of hypertension and this would limit the power of the conclusions.

2) Another limitation that might reduce the physiological relevance of the study is that conclusions are exclusively based on simulations. The approach guarantees the assessment of the relative weights of the parameters describing the compartments of the model but there is no evaluation of how realistic the overall model is because no goodness of fit was provided. In other words, the behavior of the model was not compared to real signals to understand whether the model really can describe them and can describe changes whether the baseline condition was modified.

3) The link between the model and physiological considerations reported in discussion seems to be weak. Discussion should be better linked to the model structure. After all, conclusions are mainly based on sensitivity analysis, while elucidation of mechanisms should be linked to the model structure given that modifications of model structure might alter conclusions.

4) The modification of the parameters of the model, namely the arbitrary selection of the percent variation as reported at the end of Set.2 should be more deeply commented because it might fully explain conclusions of the study. In addition to the variation of parameters the amplitude of the perturbations applied to model parameters introduced by the stochastic setting might have an important impact as well. The impact of both issues on conclusions should be more deeply commented.

Referee #2:

In this paper, the authors describe use of a previously published computational model of the cardiovascular system to identify structural and functional differences observed in hypertensive patients. They incorporated these differences

into validated parameter modifications and quantified the ability of the model to accurately represent the hypertensive state. Then the individual contribution to the hypertensive condition of the heart, the systemic arteries, the pulmonary circulation, peripheral vascular beds and the systemic venous system, were evaluated via sensitivity analysis. The primary "new" finding of these analyses was the major role played by the venous system in the determination of the hypertensive state.

A major flaw of the paper is that it does not adequately or appropriately describe and analyze previous publications on the same topic. By no means is this the first cardiovascular system model that has revealed a key role for the venous system in blood pressure regulation.

For example, 25 years ago Cavani et al (ASAIO J. 2001 Jul-Aug;47(4):377-88) published a modeling study revealing the importance of venous capacitance in dialysis induced changes in blood pressure.

Another very recent example of a modeling approach:

Yu H, Greasley PJ, Lambers Heerspink HJ, Ambery P, Ahlstrom C, Hamren B, Khan AA, Boulton DW, Hallow KM

The role of venous capacity in fluid retention with endothelin A antagonism: Mathematical modelling of the RADAR trial.

Br J Pharmacol. 2024 Nov;181(22):4693-4707

Many experimental studies also have concluded that venous compliance or capacitance play a critical role in hypertension. Just a few examples:

Samar RE, Coleman TG. Mean circulatory filling pressure and vascular compliances in the spontaneously hypertensive rat. Am J Physiol. 1979;237:H584-H589.

Martin DS, Rodrigo MC, Appelt CW.

Venous tone in the developmental stages of spontaneous hypertension.

Hypertension. 1998 Jan;31(1):139-44

Fink GD.

Arthur C. Corcoran Memorial Lecture. Sympathetic activity, vascular capacitance, and long-term regulation of arterial pressure. Hypertension. 2009 Feb;53(2):307-12

The priority of these studies needs to be recognized.

Two other topics that should be addressed in more detail:

1. Many studies reveal a change in venous capacitance in hypertension, but not venous compliance. This is touched on in the paper when covering stressed and unstressed blood volume, but could use further explication.

2. The last few sections of the Discussion don't directly address the main subject of the work and could easily be deleted.

END OF COMMENTS

Dear Editors,

We thank you and the reviewers for the careful and constructive evaluation of our manuscript. We have revised the paper extensively in response to all major concerns. In particular, we expanded the Introduction and Discussion to better acknowledge prior computational and experimental work on the role of the venous system in blood pressure regulation and hypertension, clarified the specific contribution of our modeling approach, and further discussed the distinction between venous compliance and capacitance. We also strengthened the manuscript by addressing the methodological concerns related to the hypertensive model setup, the choice of parameter variation ranges, the interpretation of the sensitivity analysis, and the limitations of the study, including the absence of direct goodness-of-fit assessment against physiological signals. Finally, we streamlined the Discussion by removing sections that were not central to the main message of the paper.

Precise details of the modifications to the manuscript, including line numbers, are provided in the replies to the reviewers, whereas the replies to the editors are more general.

We believe that these revisions have substantially improved the manuscript and we hope it is now suitable for further consideration for publication in The Journal of Physiology.

Sincerely,
Lucas O. Müller, on behalf of all authors

Senior Editor:

Thank you for submitting your manuscript to The Journal of Physiology. I have now received comments from two independent reviewers, both experts in the field, and a reviewing editor. While all see merit in your study, there are issues that will need to be addressed before we can give your manuscript further editorial consideration. I look forward to receiving your revised manuscript in due course.

Reviewing Editor:

In this study, the authors use a closed-loop computational model to perform a sensitivity analysis, concluding that reduced venous compliance is a key contributor to the hypertensive state, second only to peripheral resistance. The manuscript is well-written and the approach is interesting. However, both reviewers have raised significant concerns that must be thoroughly addressed before the manuscript can be considered further for publication.

The consensus is that the manuscript requires Major Revision. The primary concerns are outlined below:

RE.1. Insufficient context and acknowledgment of prior work.

The failure to adequately cite and discuss the extensive body of existing literature, both computational and experimental, previously identified the venous system as a key player in blood pressure regulation and hypertension needs to be acknowledged and highlighted. As noted by Referee #2, this is not the first study to highlight this role. The authors must perform a comprehensive literature review and rewrite the Introduction and Discussion:

RE.1.(a) Acknowledge and integrate prior modeling studies that have explored the role of venous capacitance/compliance.

Reply: We have incorporated experimental and modelling works suggested by the Reviewers in the revised version of the manuscript.

RE.1.(b) Discuss the significant experimental work in both animal models and humans that supports this concept (several key examples were provided by Referee #2).

Reply: We have discussed the examples provided by Referee #2, relating them to our own findings.

RE.1.(c) Clearly articulate what their specific modeling approach adds to this established field and how their quantitative findings advance our understanding beyond previous work.

Reply: We believe that the new paragraph on previous modelling added to the revised manuscript, followed by the paragraph that introduces our work, provides a clear picture on how we contribute beyond previous modelling efforts on the role of the venous system in controlling arterial pressure. In fact, previous studies did not addressed hypertension, but other states of the cardiovascular system.

RE.1.(d) Expand on the distinction between venous compliance and venous capacitance, a nuanced but important point raised by Referee #2.

Reply: Please see response to comment R2.1. We have also introduced modifications to the discussion section to clarify our modelling approach.

RE.2. Methodological justification and model limitations.

Referee #1 raised several important points regarding the study's methodology that weaken the physiological relevance of the conclusions. The authors should:

RE.2.(a) Address the critique that the model was inherently biased towards a hypertensive state. Please discuss whether the same conclusions would be reached if the analysis were performed on a model with different initial parameter settings.

Reply: Please see reply to comment R1.1.

RE.2.(b) Provide a stronger justification for the specific ranges chosen for the random parameter modifications. The authors must discuss how the arbitrary selection of these ranges might have influenced the final conclusions of the sensitivity analysis.

Reply: Please see response to comment R1.4, which resulted in modification of our discussion section in order to better elaborate on our approach.

RE.2.(c) Strengthen the link between the model's structure and the physiological mechanisms presented in the Discussion. The conclusions are derived from a sensitivity analysis, and the discussion should more explicitly reflect this specific approach.

Reply: Based on previous comments and on comments by Referees, we have removed sections of the discussion that focused on providing mechanistic explanations to experimental findings. Please see response to comment R1.3.

RE.2.(d) Acknowledge the limitation that the model was not compared against real physiological signals to provide a goodness-of-fit assessment. While performing this analysis may be beyond the scope of a revision, this limitation should be explicitly stated.

Reply: Please see response to comment R1.2.

RE.3. Focus of the Discussion.

The authors should consider the suggestion from Referee #2 to streamline the Discussion. The final sections on the revised Starling principle and haemorheology, while interesting, detract from the central message of the paper. Deleting or significantly shortening these sections could be considered.

Reply: Based on previous comments and on comments by Referees, we have removed sections of the discussion that focused on providing mechanistic explanations to experimental findings. Please see response to comment R1.3.

RE.4. Methods Details:

Perhaps - if revised, the methods require additional justification. The authors should address concerns that the model was biased toward a hypertensive state from the outset, which may limit conclusions drawn from the sensitivity analysis and provide a more thorough justification for the chosen percentage of parameter variation.

Reply: please see replies to comments R1.1, on the critique about the model being biased toward the hypertensive state and R.1.4, on the selection of parameter range. Both comments resulted in modifications to the Introduction and Discussion sections in order to clarify the raised concerns.

REFEREE COMMENTS

Referee #1:

In the study the authors utilized a previously validated model (Celant et al 2023) to model hypertension. In the present study the authors modified the parameters in a random fashion to assess the sensitivity of the model. This analysis of sensitivity allowed the authors to conclude that the reduced venous compliance might contribute significantly to hypertension in addition to the increase of peripheral resistance.

The study is interesting and well-written. However, physiological mechanisms that might be responsible for the modification of venous system in hypertensive patients remain poorly elucidated.

R1.1) One of the major limitations of the study is that the behavior of the model was biased toward a hypertensive situation. Thus, the conclusion of the study is limited to what the analysis of sensitivity has provided in this specific situation, namely the relative importance of the compartments in a situation of simulated hypertension. It is very important to check whether the same conclusions could be reached in different simulated situations and/or random setting of the mean value of parameters. My guess is that, given the structure of the model, conclusions might be the same regardless of the initial setting of hypertension and this would limit the power of the conclusions.

Reply: We believe that the Reviewer has very precisely identified the utility of our work, namely the assessment of how adaptations of the cardiovascular system observed in hypertensive patients contribute to the determination of elevated arterial blood pressure. In fact, this represents the main novel aspect of our study, since, to the best of our knowledge, such an analysis has never been performed before.

The Reviewer's concern regarding whether our conclusions could also be relevant in other situations, such as around the baseline (normotensive) scenario, has been addressed by our group in a previous paper (Müller et al., 2023). In that work, we performed a local sensitivity analysis in a cardiovascular system model and identified the venous system as a major contributor to both mean and pulse arterial pressure.

Furthermore, Reviewer 2 drew our attention to other studies in which the importance of the venous system as a major determinant of arterial pressure was also confirmed, in those cases using simpler, lumped-parameter models. We believe that these findings, together with those reported in Müller et al. (2023), strengthen the conclusions of our present work by showing that the relevance of the venous system is not limited to hypertension alone.

Since this aspect was omitted in the original version of the manuscript, we have revised the Introduction (**page 2, lines 66-73**) and Discussion (**page 14, lines 279-287**) sections to include citations of the relevant previous literature and to discuss their implications for our results.

R1.2) Another limitation that might reduce the physiological relevance of the study is that conclusions are exclusively based on simulations. The approach guarantees the assessment of the relative weights of the parameters describing the compartments of the model but there is no evaluation of how realistic the overall model is because no

goodness of fit was provided. In other words, the behavior of the model was not compared to real signals to understand whether the model really can describe them and can describe changes whether the baseline condition was modified.

Reply: We understand the Reviewer's concern regarding the validation of our modelling results. However, we would like to point out that the model has undergone continuous validation throughout its more than 10-year development history, by systematically comparing model outputs with data from the literature and, in some cases, with patient-specific measurements.

More specifically, the version of the model used in the present work was validated for both normotensive and hypertensive conditions in Celant et al. (2023). In that study, tabulated comparisons between literature data and model predictions were presented, followed by an in-depth discussion of literature evidence on hypertension and its agreement with results obtained with our model. We have now added a phrase in Section 4.1 (**page 12, lines 229-231**), referring the reader to Celant et al. (2023) for a more comprehensive discussion on the validity of the characterization of the hypertensive state by our model.

Finally, we would like to note that additional validation, in the form of comparisons between model-derived and literature haemodynamic indices, is provided in Table 2 of our manuscript.

R1.3) The link between the model and physiological considerations reported in discussion seems to be weak. Discussion should be better linked to the model structure. After all, conclusions are mainly based on sensitivity analysis, while elucidation of mechanisms should be linked to the model structure given that modifications of model structure might alter conclusions.

Reply. We agree with the Reviewer about the fact that part of our discussion was not based on the evidence provided by our modelling results. This is particularly accurate for sections 4.4 and 4.5, which have been removed. We believe that other parts of the discussion are well connected and motivated by our findings. Finally, we would like to point out that while sections 4.4 and 4.5 were not supported by our modelling results, they were an attempt to explain observations by means of physics-based principles. We have left a brief comment on both aspects (plasma and haematocrit in hypertension) in Section 4.3 (**page 15, lines 338-348**).

R1.4) The modification of the parameters of the model, namely the arbitrary selection of the percent variation as reported at the end of Set.2 should be more deeply commented because it might fully explain conclusions of the study. In addition to the variation of parameters the amplitude of the perturbations applied to model parameters introduced by the stochastic setting might have an important impact as well. The impact of both issues on conclusions should be more deeply commented.

Reply. We believe that the Reviewer refers to our description of the process that led to the stochastic simulation. For that setup, we considered a uniform distribution, centered in the literature-based variation from normo- to hypertensive and then covering a range that goes from baseline parameter to twice the literature-based perturbation. In this way our stochastic model considers uncertainty in the way we characterize hypertension, exploring the extreme

cases: a) a parameter should not be changed at all; b) literature-based variation is half of the one that should be used. If exploring this range, the sensitivity of arterial pressure does not change radically, then it is reasonable to assume that we are not missing anything important (with respect to the considered parameters). We acknowledge that this aspect did not clearly emerged from our discussion and have therefore extended the discussion section to address the Reviewer's comment (**page 13, lines 264-273**). Furthermore, we have modified the methods section to clarify our approach (**page 6, lines 167-171**).

Referee #2:

In this paper, the authors describe use of a previously published computational model of the cardiovascular system to identify structural and functional differences observed in hypertensive patients. They incorporated these differences into validated parameter modifications and quantified the ability of the model to accurately represent the hypertensive state. Then the individual contribution to the hypertensive condition of the heart, the systemic arteries, the pulmonary circulation, peripheral vascular beds and the systemic venous system, were evaluated via sensitivity analysis. The primary "new" finding of these analyses was the major role played by the venous system in the determination of the hypertensive state.

R2.0.A major flaw of the paper is that it does not adequately or appropriately describe and analyze previous publications on the same topic. By no means is this the first cardiovascular system model that has revealed a key role for the venous system in blood pressure regulation.

For example, 25 years ago Cavani et al (ASAIO J. 2001 Jul-Aug;47(4):377-88) published a modeling study revealing the importance of venous capacitance in dialysis induced changes in blood pressure.

Another very recent example of a modeling approach:

Yu H, Greasley PJ, Lambers Heerspink HJ, Ambery P, Ahlstrom C, Hamren B, Khan AA, Boulton DW, Hallow KM

The role of venous capacity in fluid retention with endothelin A antagonism: Mathematical modelling of the RADAR trial.

Br J Pharmacol. 2024 Nov;181(22):4693-4707

Many experimental studies also have concluded that venous compliance or capacitance play a critical role in hypertension. Just a few examples:

Samar RE, Coleman TG. Mean circulatory filling pressure and vascular compliances in the spontaneously hypertensive rat. Am J Physiol. 1979;237:H584-H589.

Martin DS, Rodrigo MC, Appelt CW.

Venous tone in the developmental stages of spontaneous hypertension.

Hypertension. 1998 Jan;31(1):139-44

Fink GD.

Arthur C. Corcoran Memorial Lecture. Sympathetic activity, vascular capacitance, and long-term regulation of arterial pressure. Hypertension. 2009 Feb;53(2):307-12

The priority of these studies needs to be recognized.

Reply. We thank the Reviewer for pointing out missing relevant works in our literature revision. We have included them in relevant points of the manuscript, in particular in the introduction (**page 2, lines 66-73**) and discussion (**page 14, lines 279-287**) sections. We believe that these references strengthen the conclusions of our work, offering complementary methodological/modelling results and at the same time widening the clinical evidence of the role of the venous system in hypertension.

Two other topics that should be addressed in more detail:

R2.1. Many studies reveal a change in venous capacitance in hypertension, but not venous compliance. This is touched on in the paper when covering stressed and unstressed blood volume, but could use further explication.

Reply. Compliance and capacitance are e**REFERENCES**xtremely intertwined concepts. Please see schematic reported in Figure 1, where it becomes clear how changes in one parameter or the other have similar impact on pressure (or volume, depending on the experiment). In the cardiovascular system, veins are not isolated, so changes in compliance or unstressed volumes would both result i**REFERENCES**n reduced capacitance in the sense that for a fixed pressure, less volume can be accommodated. To experimentally separate changes due to either player (V0 and C) is rather complicated, especially if we consider human subjects. Since many experimental studies on humans found a reduced total effective compliance, and compliance is mainly due to systemic venous compliance, we decided to act on this model parameter as a representative effective parameter on observed changes in hypertensive patients. The name given by experimental studies contains “effective”, implying that compliance is chosen as an effective parameter where most probably both aspects contribute to the reduction of the effective parameter: a reduction of unstressed volumes consequent to stiffening of the veins. We have extended the discussion of the revised manuscript to better explain our modelling choice (**page 14, lines 299-309**).

Figure 1: schematic on the impact of changes in unstressed volume and compliance on pressure for fixed volume.

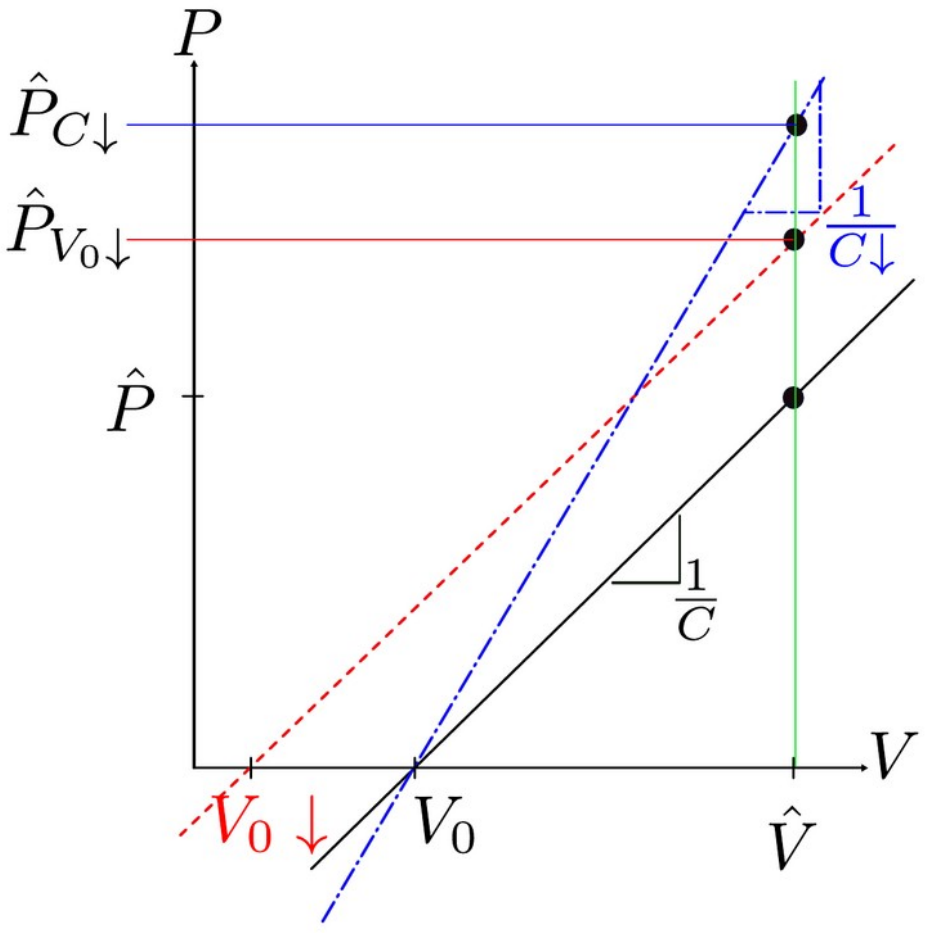

R2.2. The last few sections of the Discussion don't directly address the main subject of the work and could easily be deleted.

Reply. Sections 4.4 and 4.5 have been removed. Please see reply to comment R1.3.

REFERENCES

Celant M, Toro EF, Bertaglia G, Cozzio S, Caleffi V, Valiani A, Blanco PJ, Müller LO. Modeling essential hypertension with a closed-loop mathematical model for the entire human circulation. Int J Numer Method Biomed Eng. 2023 Nov;39(11):e3748. doi: 10.1002/cnm.3748. Epub 2023 Jul 5. PMID: 37408358.

Müller LO, Watanabe SM, Toro EF, Feijóo RA, Blanco PJ. An anatomically detailed arterial-venous network model. Cerebral and coronary circulation. Front Physiol. 2023 Jun 26;14:1162391. doi: 10.3389/fphys.2023.1162391. PMID: 37435309; PMCID: PMC10332167.

Dear Associate Professor Müller,

Re: JP-RP-2026-289895R1 "Unveiling the role of the venous system in arterial hypertension: A computational study" by Eleuterio F. Toro, Lucas Omar Müller, Tonja W Emans, Fiona D McBryde, and Julian F. R. Paton

We are pleased to tell you that your paper has been accepted for publication in The Journal of Physiology.

____

IMPORTANT: Before publication, please can you confirm the status of the figures relative to the book publication you advised. Should the figures be acknowledged as previously published in the book? Additionally, we have received multiple copies of each figure. It is not clear which should be used. Please confirm.

____

The last Word (or similar) version of the manuscript provided will be used by the Production Editor to prepare your proof. When this is ready you will receive an email containing a link to Wiley's Online Proofing System. The proof should be thoroughly checked and corrected as promptly as possible.

Authors should note that it is too late at this point to offer corrections prior to proofreading. Major corrections at proof stage, such as changes to figures, will be referred to the Editors for approval before they can be incorporated. Only minor changes, such as to style and consistency, should be made at proof stage. Changes that need to be made after proof stage will usually require a formal correction notice.

All queries at proof stage should be sent to: TJP@wiley.com.

Following copyediting and typesetting the accepted version will be published online.

Yours sincerely,

Vaughan Macefield
Senior Editor
The Journal of Physiology

IMPORTANT POINTS TO NOTE FOLLOWING ACCEPTANCE OF YOUR PAPER:

• IMPORTANT NOTICE ABOUT OPEN ACCESS: To assist authors whose funding agencies mandate immediate public access to published research findings, The Journal of Physiology allows authors to pay an Open Access (OA) fee to have their papers made freely available immediately on publication.

The Corresponding Author will receive an email from Wiley with details on how to register or log in to Wiley Authors where you will be able to place an order.

• You can check if your funder or institution has a Wiley Open Access Account here:
https://authors.wiley.com/author-resources/Journal-Authors/open-access/author-compliance-tool.html

• TRANSPARENT PEER REVIEW POLICY: To improve the transparency of its peer review process, The Journal of Physiology publishes online as supporting information the peer review history of all articles accepted for publication. Readers will have access to decision letters, including Editors' comments and referee reports, for each version of the manuscript as well as any author responses to peer review comments. Referees can decide whether or not they wish to be named on the peer review history document.

• You can help your research get the attention it deserves! Check out Wiley's free Promotion Guide for best-practice recommendations for promoting your work at: www.wileyauthors.com/eeo/guide. You can learn more about Wiley Editing Services which offers professional video, design, and writing services to create shareable video abstracts, infographics, conference posters, lay summaries, and research news stories for your research at: www.wileyauthors.com/eeo/promotion.

• If you would like to receive our 'Research Roundup', a monthly newsletter highlighting the cutting-edge research published in The Physiological Society's family of journals (The Journal of Physiology, Experimental Physiology, Physiological Reports, The Journal of Nutritional Physiology and The Journal of Precision Medicine: Health and Disease), please click this link, fill in your name and email address and select 'Research Roundup':
https://www.physoc.org/journals-and-media/membernews

EDITOR COMMENTS

Reviewing Editor:

Thank you for submitting your revised manuscript. Both referees and myself agree that your revisions have significantly improved the focus, clarity, and impact of the paper.

Senior Editor:

Thank you for submitting your revised manuscript to The Journal of Physiology. As you will see, both reviewers and the Reviewing Editor are satisfied with your amendments. Accordingly, I am pleased to report that your manuscript is now considered acceptable for publication.

REFEREE COMMENTS

Referee #1:

The manuscript has been substantially improved. The authors replied satisfactorily to all my issues and took into account the suggestions given. I have no additional comments.

Referee #2:

Your revision of the original submission produced a much clearer and compelling version. The findings are important and thought-provoking in strongly challenging the current paradigm used to explain the pathogenesis of hypertension.
